# Case Report: Imaging features of fetal intracranial immature teratoma

**DOI:** 10.3389/fonc.2026.1714419

**Published:** 2026-03-19

**Authors:** Tingting Cai, Wenwen Zhang, Linghong Qi, Qiongshan Li, Mingsong Liu, Xue Ping Shen, Zhi Li

**Affiliations:** 1 Department of Ultrasound, Huzhou Maternity & Child Health Care Hospital, Huzhou, China; 2 Department of Pathology, Huzhou Maternity & Child Health Care Hospital, Huzhou, China; 3 Department of Radiology, Huzhou Maternity & Child Health Care Hospital, Huzhou, China; 4 Department of Obstetrics, Huzhou Maternity & Child Health Care Hospital, Huzhou, China; 5 Department of Medical Laboratory Center, Huzhou Maternity & Child Health Care Hospital, Huzhou, China

**Keywords:** case report, congenital brain tumor, fetus, immature teratoma, magnetic resonance imaging

## Abstract

Fetal intracranial immature teratomas are exceedingly rare. In most cases, the exact site of origin cannot be determined. Early and accurate diagnosis plays a pivotal role in pregnancy management and delivery planning, making imaging evaluation particularly important. However, prenatal imaging diagnosis remains challenging. We report a case of fetal immature teratoma confirmed by autopsy pathology, with comprehensive prenatal and post-induction imaging examinations. Prenatal ultrasound revealed a mixed echogenic mass at the base of the fetal skull with multiple hyperechoic foci, and CDFI demonstrated relatively abundant internal vascular signals. Prenatal MRI demonstrated a mass-like intracranial lesion with heterogeneous signal intensity, closely related anteriorly to the middle cranial fossa. The adjacent brain parenchyma was compressed and displaced, with indistinct cerebral sulci and gyri and thinning of the cerebral cortex. Post-induction MRI showed partial protrusion of the intracranial lesion into the left lateral ventricle, without evident fat signal. Post-induction CT further demonstrated multiple calcifications within the portion of the lesion extending into the left lateral ventricle. Prenatal ultrasound can detect calcifications and vascularity within fetal intracranial immature teratomas, whereas prenatal MRI offers superior lesion localization, overall visualization, delineation of relationships with adjacent brain structures, and evaluation of invasion extent. The complementary use of both modalities provides significant advantages in improving diagnostic accuracy.

## Introduction

Fetal brain tumors are rare, with teratomas being the most common histological subtype, accounting for up to 50% of cases and predominantly occurring in the supratentorial region (1). Fetal teratomas can develop at various anatomical sites, most frequently in the sacrococcygeal region, but also in the head and neck, thoracic cavity, retroperitoneum, mediastinum, intracranial region, and gonads (2). Fetal intracranial teratomas are exceedingly rare (3) and can originate from the pituitary gland, suprasellar region, third ventricle, or cerebral hemispheres. In the majority of cases, the precise site of origin remains indeterminate (4–6). According to the World Health Organization classification of central nervous system tumors, teratomas are classified into mature teratomas, immature teratomas, and teratomas with malignant transformation (7). Reports of fetal intracranial immature teratoma are relatively rare (3, 8–11), and most published studies have described only a single prenatal imaging modality. In the present report, we describe a case of fetal immature teratoma evaluated using comprehensive multimodal imaging, including prenatal ultrasound, prenatal magnetic resonance imaging (MRI), post-induction MRI, and post-induction computed tomography (CT). The imaging findings and differential diagnoses are analyzed to improve understanding of this condition and to enhance diagnostic accuracy.

## Case report

The patient was a 28-year-old woman, at 23 weeks and 5 days of gestation. She reported no abdominal pain, distension, vaginal bleeding, or fluid leakage. On August 13, 2024, prenatal ultrasonography performed at an outside hospital revealed bilateral lateral ventricular dilatation and a medium-echo intracranial mass in the fetus, prompting referral to our hospital for further evaluation. The patient had not received routine prenatal care during this pregnancy. She had no history of hypertension, diabetes, chronic illnesses, infectious diseases, or long-term medication use. She reported no known food or drug allergies. She reported no history of exposure to toxic substances or ionizing radiation, and denied smoking, alcohol consumption, or other harmful lifestyle behaviors. She reported no family history of hereditary disorders or infectious diseases. The timeline of imaging examinations and the corresponding diagnoses are presented in **Table 1**.

**Table 1:** The timeline of imaging examinations and the corresponding diagnoses.

Timeline	Inspection method	Diagnosis result
2024.8.13	Prenatal ultrasonography	Heterogeneous intracranial mass, suspicious for malignancy
2024.8.27	Prenatal MRI	Malignant intracranial tumor, bilateral lateral ventricular dilatation, and cortical dysplasia
2024.9.1	Post-termination MRI	Intracranial mass suggestive of teratoma and cortical dysplasia
2024.9.1	Post-termination CT	Intracranial mass suggestive of teratoma and cortical dysplasia
2024.10.10	Autopsy	Intracranial immature teratoma

Ultrasound: Ultrasound examination was performed using a GE Voluson E8 color Doppler system (GE Healthcare, USA) equipped with an RAB6-D transducer with a frequency range of 1–5 MHz.

MRI: MRI was performed on a 1.5-T Siemens Avanto scanner (Siemens Healthineers, Germany) using an abdominal coil. The routine slice thickness was 3–4 mm with an interslice gap of 0.6 mm, a field of view (FOV) of 380–400 mm, and 1–2 signal averages. Imaging sequences included true fast imaging with steady-state precession (TrueFISP), half-Fourier acquisition single-shot turbo spin-echo (HASTE), and two-dimensional T1-weighted fast low-angle shot imaging (2D Turbo FLASH, TFL).

CT: Cranial CT was performed using a GE Optima CT540 16-slice scanner (GE Healthcare, USA). The scanning parameters were as follows: tube voltage 80 kV, tube current 10 mA, pitch 1.0, and slice thickness 5 mm.

Chromosomal microarray analysis: Amniotic fluid cells were cultured *in situ* for chromosomal microarray analysis.

Trio whole-exome sequencing (Trio-WES):Libraries were prepared using the Twist Library Preparation Enzymatic Fragmentation Kit 2.0 (Twist Bioscience, USA), and target regions were captured using Twist hybridization capture reagents. Following magnetic bead purification, library quality control was performed, requiring a library concentration ≥10 ng/μL and an average fragment size of 300–400 bp. Qualified libraries were sequenced on an MGI DNBSEQ-T7 platform (MGI Tech, China) using 2 × 150 bp paired-end sequencing.

**Figure 1:**
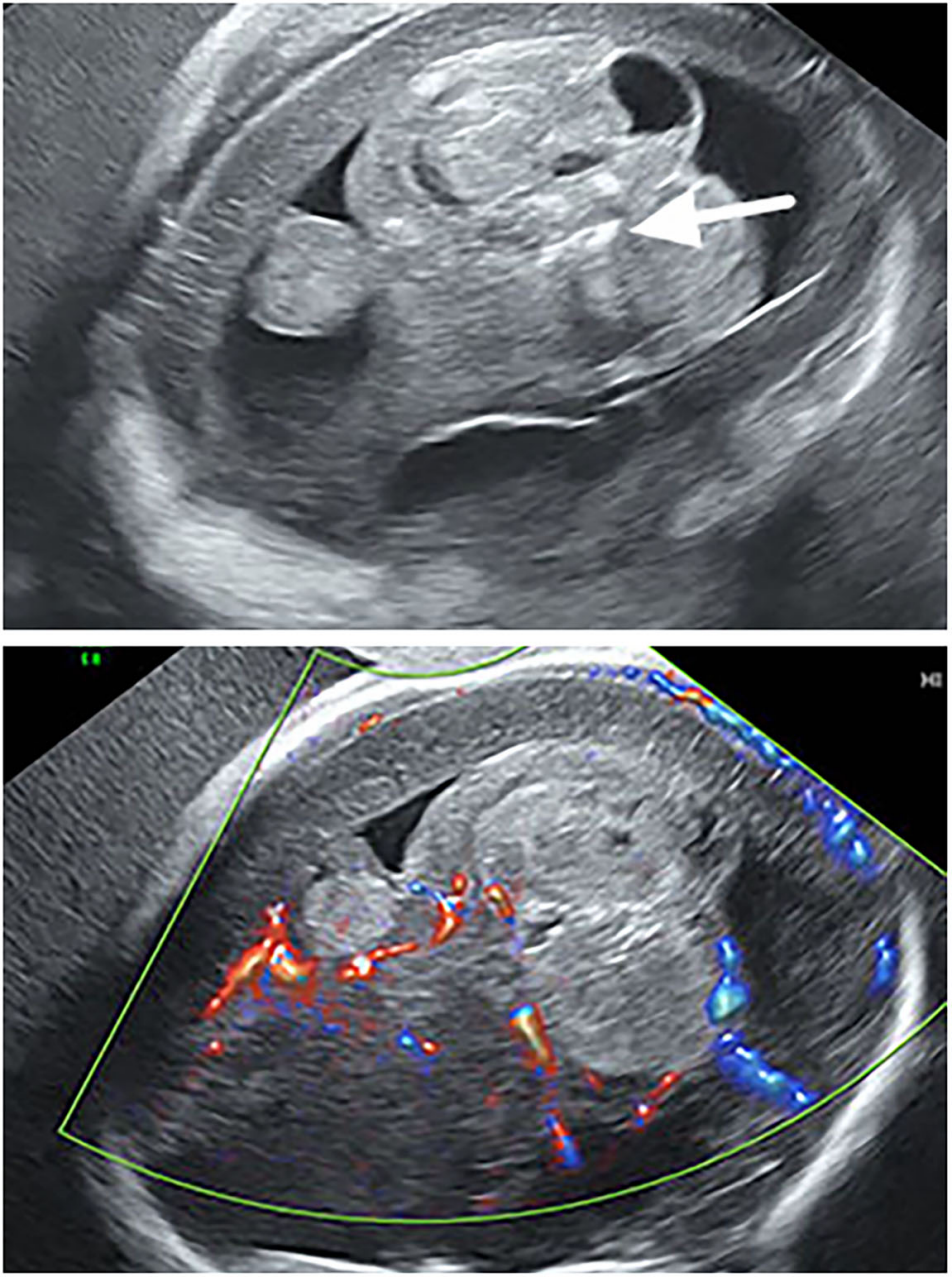
Prenatal ultrasound findings. **(A)** The intracranial mass contains multiple hyperechoic foci (long arrow). **(B)** CDFI demonstrates relatively abundant intralesional vascularity.

**Figure 2:**
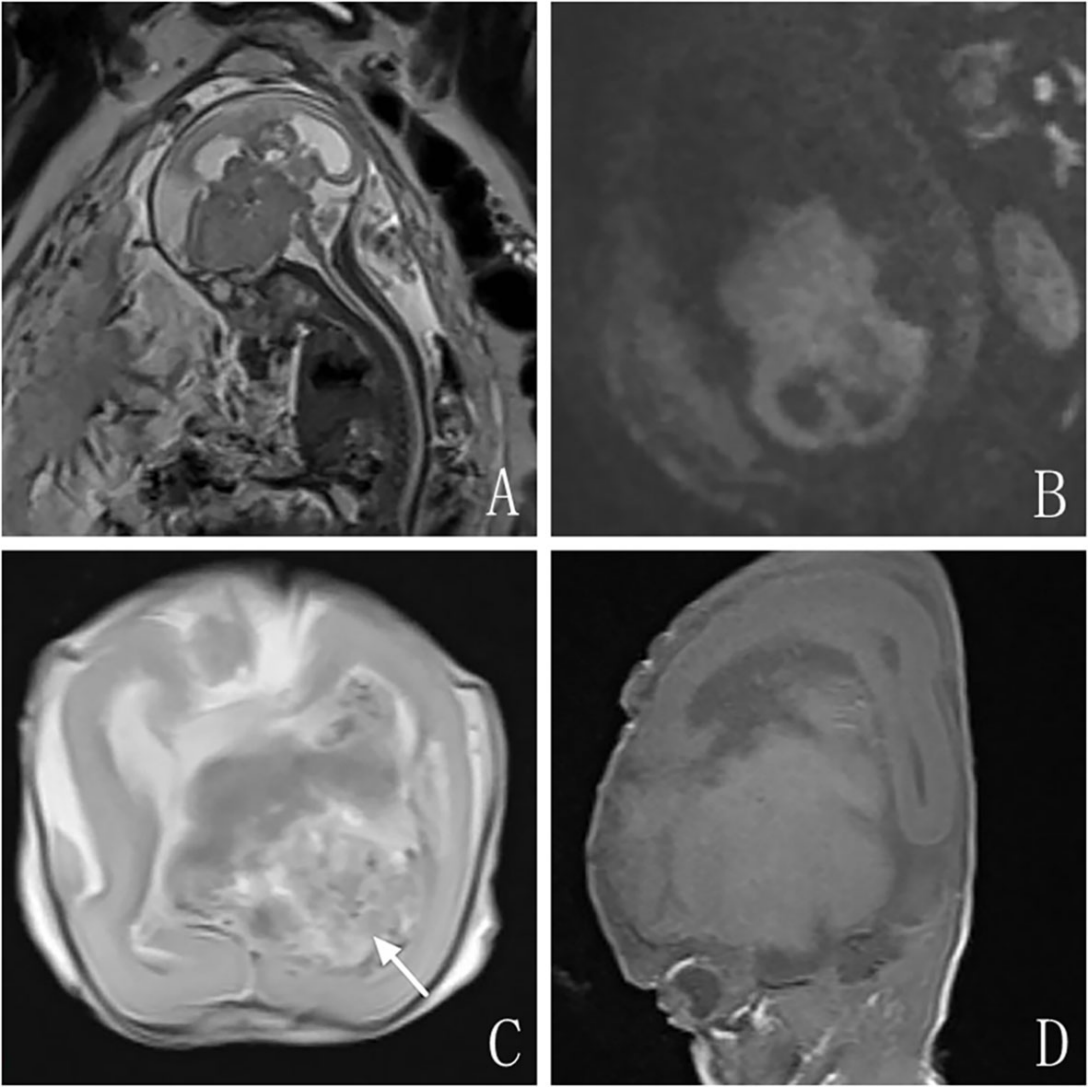
Prenatal and post-termination MRI findings. **(A)** Sagittal T2WI demonstrating the lesion at the anterior cranial base, closely related to the middle cranial fossa (long arrow), extending posteriorly from the midline, with slightly hyperintense signal. **(B)** Axial DWI (b = 700) showing the lesion with isointense signal, similar to normal brain parenchyma. **(C)** Post-termination axial T2WI showing the lesion protruding into the left lateral ventricle with heterogeneous signal intensity (long arrow). **(D)** Post-termination sagittal T1WI demonstrating posterior and superior displacement of normal brain parenchyma due to mass effect.

Prenatal ultrasonography revealed a heterogeneous echogenic mass located at the fetal skull base, extending toward the cranial vault, measuring approximately 85 × 66 × 56 mm. The lesion was irregular in shape, with relatively well-defined margins, heterogeneous internal echogenicity, and multiple hyperechoic foci (**Figure 1A**). Color Doppler flow imaging (CDFI) demonstrated abundant vascularity within the lesion (**Figure 1B**). Both cerebral hemispheres were compressed against the cranial vault, with poorly visualized cortical sulci and bilateral lateral ventricular dilatation.Prenatal MRI demonstrated a lobulated intracranial mass, with its anterior margin closely abutting the middle cranial fossa and extending posteriorly from the midline of the anterior cranial base (**Figure 2A**). The lesion measured approximately 8.4 × 6.4 × 5.4 cm, was predominantly solid with an irregular configuration, demonstrated isointensity on T1-weighted imaging (T1WI), slight hyperintensity on T2-weighted imaging (T2WI), and isointensity on diffusion-weighted imaging (DWI) (**Figure 2B**). A portion of the lesion extended into the left lateral ventricle, exhibiting a slightly heterogeneous signal on T2WI.The cerebral parenchyma was compressed and displaced, with poorly visualized cortical sulci and cortical thinning. Both lateral ventricles were dilated, with maximal widths of approximately 1.5 cm on the right and 1.2 cm on the left.

**Figure 3:**
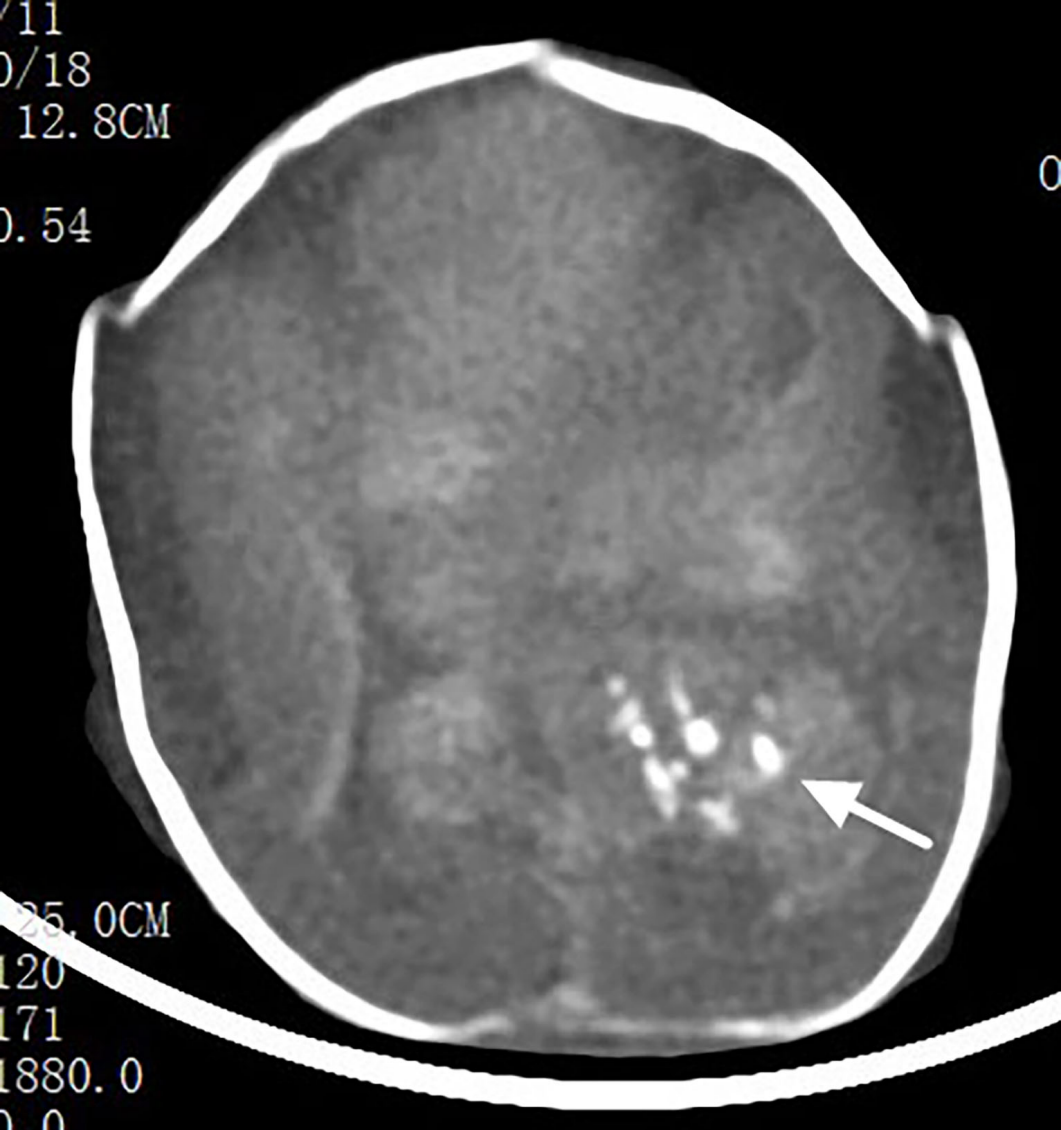
Post-termination CT image demonstrating. Scattered intralesional calcifications within the portion of the mass protruding into the left lateral ventricle (long arrow).

Post-termination MRI demonstrated an irregular intracranial mass, partially extending into the left lateral ventricle, exhibiting mixed signal intensity on T1WI and T2WI (**Figure 2C**). The remaining portions of the lesion demonstrated signal intensities similar to the cerebral parenchyma on T1WI and T2WI, with no apparent fat signal, and appeared isointense on DWI. The cerebral parenchyma was markedly compressed and displaced in a posterior-superior direction (**Figure 2D**), with poorly visualized cortical sulci. Post-termination CT demonstrated a lobulated intracranial mass with an irregular contour, partially protruding into the left lateral ventricle, containing multiple intralesional calcifications (**Figure 3**). The cerebral parenchyma was compressed and displaced, with indistinct cortical sulci. Fetal chromosomal analysis and whole-exome sequencing were both negative.

**Figure 4:**
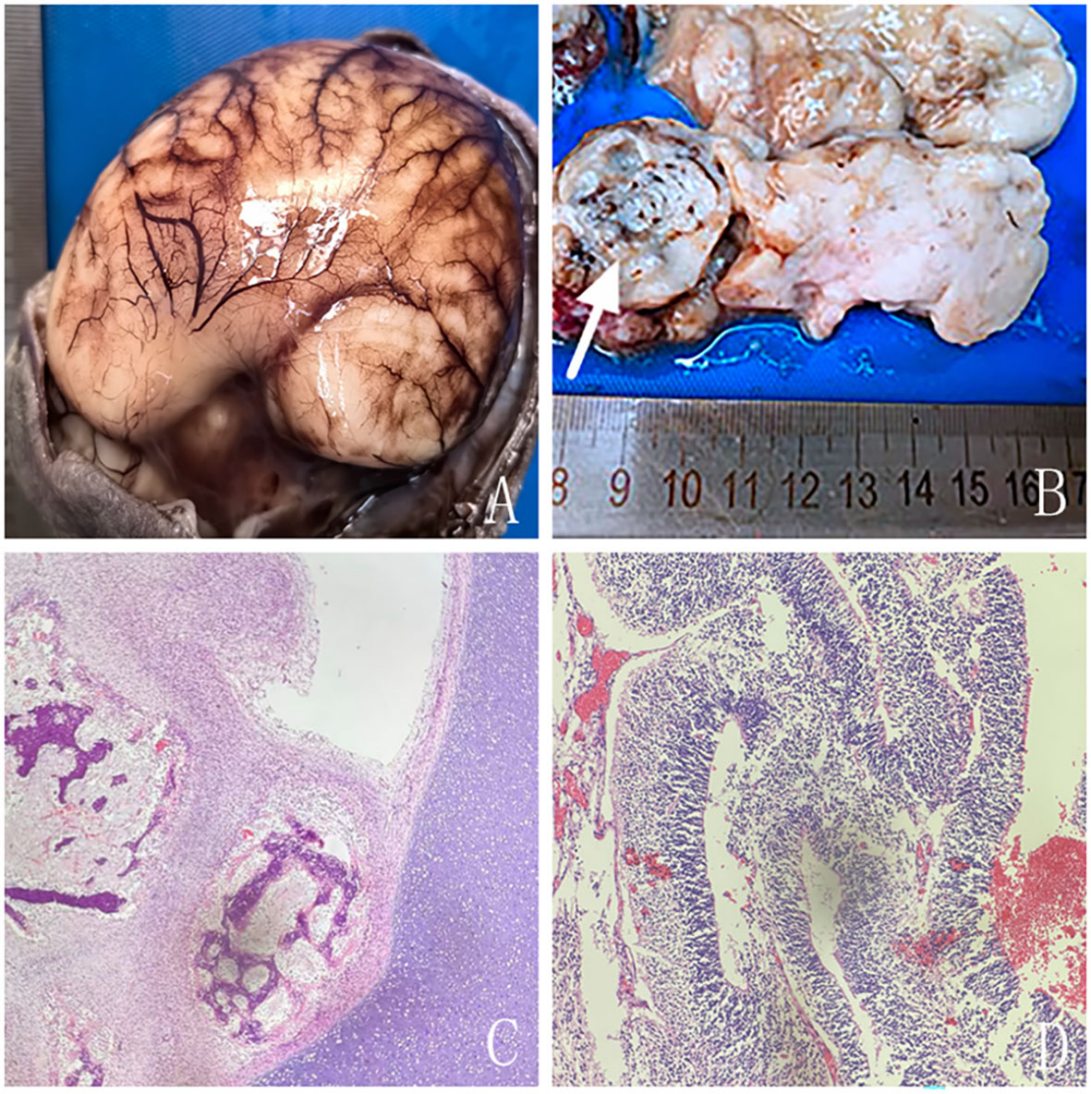
Pathological findings. **(A)** The brain surface was tense with markedly shallow sulci and reduced gyri. **(B)** Gross specimen showing a predominantly gray-white tumor with firm regions (long arrow) and areas of fish-flesh–like, friable tissue (arrow). **(C)** Hematoxylin–eosin (HE) staining, original magnification ×40, demonstrating mature bone and cartilage tissue. **(D)** HE staining, original magnification ×100, revealing immature neural tissue.

Autopsy demonstrated a tense cranial vault with markedly effaced cortical sulci (**Figure 4A**). A longitudinal mass extended from the frontal region to the middle cranial fossa, spanning the anterior to middle cranial compartments. The frontal aspect of the mass was well-circumscribed, whereas the margin adjacent to the middle cranial fossa was indistinct, suggestive of involvement of the sphenoid bone. The mass caused bilateral compression of the lateral ventricles. On gross sectioning, the majority of the lesion consisted of soft gray-white tissue, interspersed with areas of gray-red tissue. Multiple calcified foci were identified within the mass (**Figure 4B**).Histopathological examination confirmed the diagnosis of an intracranial immature teratoma (**Figures 4C, D**).

## Discussion

The etiology of fetal intracranial teratomas remains poorly understood. Current evidence indicates that abnormal germ cell differentiation during gestational weeks 3–5 may lead to the formation of ectopic pluripotent cells, which can subsequently differentiate or undergo malignant transformation. The pathogenesis of fetal immature teratoma is complex and remains incompletely understood. It is generally believed to involve abnormalities in early embryonic development, particularly impaired differentiation of primordial germ cells. These cells may remain arrested at an immature embryonic stage and subsequently undergo aberrant proliferation, ultimately leading to tumor formation. Early and accurate diagnosis of fetal tumors is critical for optimal pregnancy management and delivery planning (10).However, detection of fetal intracranial teratomas during the first trimester is exceedingly rare, thereby posing a significant challenge for early diagnosis. Congenital tumors frequently differ from later-onset tumors in terms of histology, pathophysiology, and imaging characteristics, further complicating accurate diagnosis (12–14).

Prenatal ultrasonography, owing to its wide availability and moderate cost, is generally considered the first-line modality for diagnosing fetal intracranial teratomas. Ultrasonographic findings can be classified into direct and indirect features. Direct features represent the intrinsic imaging characteristics of the tumor, primarily presenting as mixed cystic-solid or solid echogenic masses. Indirect features correspond to secondary alterations induced by tumor growth and compression, including midline shift, ventricular compression, ventricular dilatation, and, in severe cases, hydrocephalus. In this case, the direct features were demonstrated by a mixed echogenic mass located at the fetal cranial base, exhibiting an irregular shape and partially heterogeneous internal echoes, with multiple hyperechoic foci. The indirect features included compression of the cerebral parenchyma toward the cranial vault, poorly visualized gyri and sulci, and bilateral lateral ventricular dilatation. Previous reports indicate that fetal intracranial teratomas may compress the brainstem, impairing fetal swallowing, with indirect signs such as polyhydramnios (3); in severe cases, fetal hydrops can develop. Approximately 7.8% of cases are associated with additional structural anomalies, including hypertelorism, micrognathia, or horseshoe kidney (15). In this case, no indirect signs of polyhydramnios were observed, and autopsy did not identify the aforementioned structural anomalies. Teratomas frequently exhibit calcifications, although such features are uncommon in immature teratomas. In this case, prenatal ultrasonography identified calcification within the lesion extending into the left lateral ventricle, which was subsequently confirmed by post-termination CT imaging and autopsy.

MRI serves as an important adjunct to ultrasonography in the diagnosis of fetal intracranial tumors. Milani et al. (12) reported that MRI provides superior depiction of tissue density, is not influenced by the cranial bone halo, and clearly delineates the relationship between the tumor and adjacent brain structures, which is highly valuable for identifying tissue architecture and precise tumor localization. Xia et al. (16) reported that MRI can detect small lesions, such as focal hemorrhage and periventricular nodules, as well as intracranial masses that may be missed on ultrasonography. Moreover, MRI provides accurate delineation of tumor extent and clear visualization of surrounding anatomical structures, which is essential for evaluating the degree of lesion invasion (17). In this case, prenatal MRI demonstrated that the anterior margin of the lesion was closely associated with the middle cranial fossa, extending upward and posteriorly, with dorsal and superior displacement of the brain parenchyma due to compression, suggesting a middle cranial fossa origin. MRI is relatively insensitive to the detection of calcifications. In this case, prenatal MRI demonstrated slightly heterogeneous signal intensity within the portion of the lesion protruding into the left lateral ventricle, indirectly suggesting the presence of calcifications. Postnatal CT subsequently confirmed multiple intralesional calcifications. Congenital immature teratomas differ from those arising later in life, as congenital lesions do not necessarily contain adipose components. In this case, both prenatal MRI and post-termination CT revealed no adipose components, which was subsequently confirmed by autopsy. Immature teratomas are typically solid and demonstrate rapid growth, potentially leading to necrosis or hemorrhage (18, 19). None of the imaging examinations in this case demonstrated evidence of necrosis or hemorrhage. This absence may be related to the gestational age of 23 weeks (mid-second trimester), at which time the lesion may not yet have progressed sufficiently to develop necrosis or hemorrhage. Prenatal ultrasonography and post-termination CT confirmed calcification within the lesion, whereas the remaining tumor comprised immature neuroepithelial components. Consequently, prenatal MRI signal characteristics and post-termination CT appearances resembled those of normal brain tissue. When prenatal ultrasound reveals an irregular midline intracranial mass accompanied by calcifications and abundant vascularity on CDFI, and prenatal MRI demonstrates invasion of adjacent brain tissue with significant compression and displacement, the possibility of an immature teratoma should be considered.

Fetal intracranial immature teratomas are exceedingly rare and diagnostically challenging, and should be differentiated from the following entities: ①Astrocytoma: Astrocytomas typically present as large hemispheric masses that displace midline structures and may cause obstructive hydrocephalus with progressive head enlargement (20, 21). They generally lack marked aggressive features and most commonly occur after 32 weeks of gestation, which is later than most other congenital brain tumors (22). ②Choroid plexus papilloma: Choroid plexus papillomas are solid intraventricular tumors frequently associated with marked hydrocephalus. On ultrasound, they typically appear as hyperechoic masses within a dilated lateral ventricle (18). The site of origin is an important diagnostic clue. These tumors often demonstrate a lobulated morphology with abundant internal vascularity on Doppler imaging (1). ③Ependymoma: Ependymomas account for approximately 2–3% of congenital brain tumors (10). They arise from undifferentiated neuroepithelial cells and commonly present as large, multilobulated masses located either supratentorially or infratentorially. Calcifications may be present, and extension into the posterior fossa or spinal canal can occur. In contrast, fetal immature teratomas are typically irregular in morphology and predominantly supratentorial, making lesion shape and location helpful features for differential diagnosis.④Craniopharyngioma: Craniopharyngiomas are benign tumors arising from the sellar or suprasellar region and are rare in the perinatal period (23). Imaging usually demonstrates a large mass that may be difficult to distinguish from an immature teratoma. MRI is valuable for assessing residual brain structures and precisely defining tumor location. Large tumor size may lead to increased head circumference, and secondary obstruction of cerebrospinal fluid pathways can result in hydrocephalus (18).

In this case, prenatal MRI and post-termination CT both revealed compression and displacement of the cerebral parenchyma, poorly delineated sulci, and cortical thinning. Autopsy further demonstrated markedly shallow sulci and reduced gyri, consistent with fetal cortical dysplasia. Previous studies have rarely reported an association between fetal intracranial teratomas and chromosomal abnormalities. In the present case, both chromosomal microarray analysis and whole-exome sequencing yielded negative results. As no pathogenic or likely pathogenic variants were identified, further parental genetic testing was not pursued. Thus, whether the fetal cortical dysplasia observed in this case is related to the immature teratoma warrants further investigation.

Tumor size, location, and pathological characteristics are key factors influencing prognosis. Fetal immature teratomas tend to grow rapidly and may invade, compress, and destroy adjacent normal brain tissue, contributing to a high mortality rate. Management strategies vary across regions. In many prenatally diagnosed cases, therapeutic termination of pregnancy is considered, whereas in selected cases continuation of pregnancy followed by cesarean delivery has been reported. Successful surgical resection of neonatal intracranial immature teratomas has been described (24). However, large-scale studies and long-term follow-up data remain limited, and intrauterine therapeutic interventions are still at an exploratory stage (25). For prenatally detected cases, combined prenatal ultrasound and MRI are recommended to comprehensively evaluate tumor characteristics and associated complications. Detailed prenatal counseling should be provided based on imaging findings, taking into account gestational age at diagnosis, disease severity, and parental preferences to guide individualized management decisions. If pregnancy continuation is chosen, serial imaging is recommended to monitor tumor progression. Multidisciplinary consultation—including neonatology, neurosurgery, plastic surgery, otolaryngology, and neonatal intensive care—should be arranged in advance to develop an appropriate perinatal management and delivery plan.

## Conclusion

Fetal intracranial immature teratoma is an extremely rare condition and presents substantial diagnostic challenges. Prenatal ultrasound is valuable for detecting intralesional calcifications and vascularity, whereas prenatal MRI provides superior assessment of lesion localization, overall extent, involvement of adjacent brain structures, and degree of invasion. The complementary use of these two imaging modalities enhances diagnostic accuracy. Once identified, management strategies should be individualized based on imaging findings and clinical circumstances.

## Data Availability

The original contributions presented in the study are included in the article/supplementary material. Further inquiries can be directed to the corresponding authors.
